# Supplementary material for: Centriolar satellites are required for efficient ciliogenesis and ciliary content regulation
Source: EMBO Rep. 2019 Apr 25;20(6):e47723. doi: 10.15252/embr.201947723 (PMC6549029; doi:10.15252/embr.201947723)
Supplement: Supplementary file 1 — Appendix [file EMBR-20-e47723-s001.pdf]

Table of Contents

Appendix Figure S1. Expression of myc-PCM1 in IMCD3 PCM1 KO cells

Appendix Figure S1

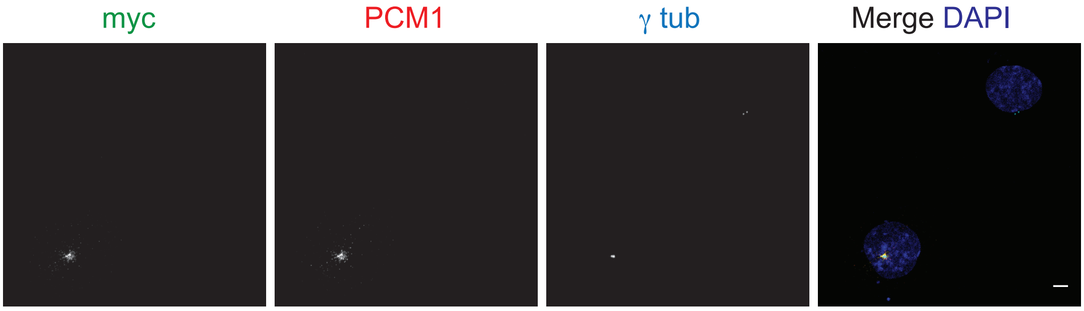

Appendix Figure S1. Expression of myc-PCM1 in IMCD3 PCM1 KO cells

Control and IMCD3 KO cells were transfected with myc-BirA*-PCM1, fixed and stained with antibodies against myc, PCM1, gamma-tubulin. DNA was stained with DAPI. Scale bar, 5 µm.
