# Supplementary material for: Centriolar satellites are required for efficient ciliogenesis and ciliary content regulation
Source: EMBO Rep. 2019 Apr 25;20(6):e47723. doi: 10.15252/embr.201947723 (PMC6549029; doi:10.15252/embr.201947723)
Supplement: Supplementary file 2 — Expanded View Figures PDF [file EMBR-20-e47723-s002.pdf]

Expanded View Figures

Figure EV1. IMCD3 PCM1 KO cells are devoid of satellite structures.

A IMCD3 PCM1 KO clones are all compound heterozygotes with mutations that lead to early stop codons. 1,000-bp region around the gRNA-target site was PCR-amplified and cloned. Sequencing of five different clones for each line identified one-nucleotide (nt) deletion on one allele and one-nt insertion on the other for line 1, 16-nt deletion on one allele and 2-nt insertion on the other for line 2, and 16-nt deletion for one allele and 4-nt deletion on the other for line 3.

B Translation products on protein-coding exon 2 of the gRNA-targeting exon in IMCD3 KO clones.

C Immunofluorescence analysis of control and IMCD3 PCM1 KO clones. Cells were fixed and stained for centrosomes with anti-γ-tubulin antibody and PCM1 with PCM1-N antibody (targeting 1–254 amino acids) and PCM1-C antibody (targeting 1,665–2,026 amino acids). Scale bar, 4 μm.

D FACS sorting of propidium iodide-stained IMCD3 control and PCM1 KO cells. Graphs are prepared with the cell number on *y*-axis and PI fluorescence reflecting the DNA content on *x*-axis.

© 2019 The Authors

A IMCD3 WT: AAGATTCCACATACGTTTCCACACAGCAGATATATGAC

KO1 allele 1: AAGATT CCACAT- CGTTTCCACACAGCAGATATATGAC
KO1 allele 2: AAGATTTCCACATACGTTTCCACACAGCAGATATATGAC

KO2 allele 1: AAGATTCCACATA---------------------------------ATATATGAC
KO2 allele 2: AAGATTCCACATATACGTTTCCACACAGCAGATATATGAC

KO3 allele 1: AAGATTCCACATA---------------------------------ATATATGAC
KO3 allele 2: AAGATTCCACAT--------TTCCACACAGCAGATATATGAC

---- deletion
TA insertion

B Mouse PCM1 Exon 3 translation (Coding exon 2)

IMCD3 WT: EWGGQQKKANRSSEKNKKKFGVASDKRVTNAISPESSPGVGRRRTKIPHTFPHSRYMTQMSVPEQAELEKLKQRINFSDLDQ

KO1 allele 1: EWGGQQKKANRSSEKNKKKFGVASDKRVTNAISPESSPGVGRRRTKIPHRFHTADI*
KO1 allele 2: EWGGQQKKANRSSEKNKKKFGVASDKRVTNAISPESSPGVGRRRTKISTYVSTQQIYDSDVCSRAGRTRET*

KO2 allele 1: EWGGQQKKANRSSEKNKKKFGVASDKRVTNAISPESSPGVGRRRTKIPHNI*
KO2 allele 2: EWGGQQKKANRSSEKNKKKFGVASDKRVTNAISPESSPGVGRRRTKIPHIRFHTADI*

KO3 allele 1: EWGGQQKKANRSSEKNKKKFGVASDKRVTNAISPESSPGVGRRRTKIPHNI*
KO3 allele 2: EWGGQQKKANRSSEKNKKKFGVASDKRVTNAISPESSPGVGRRRTKIPHFHTADI*

* stop codon

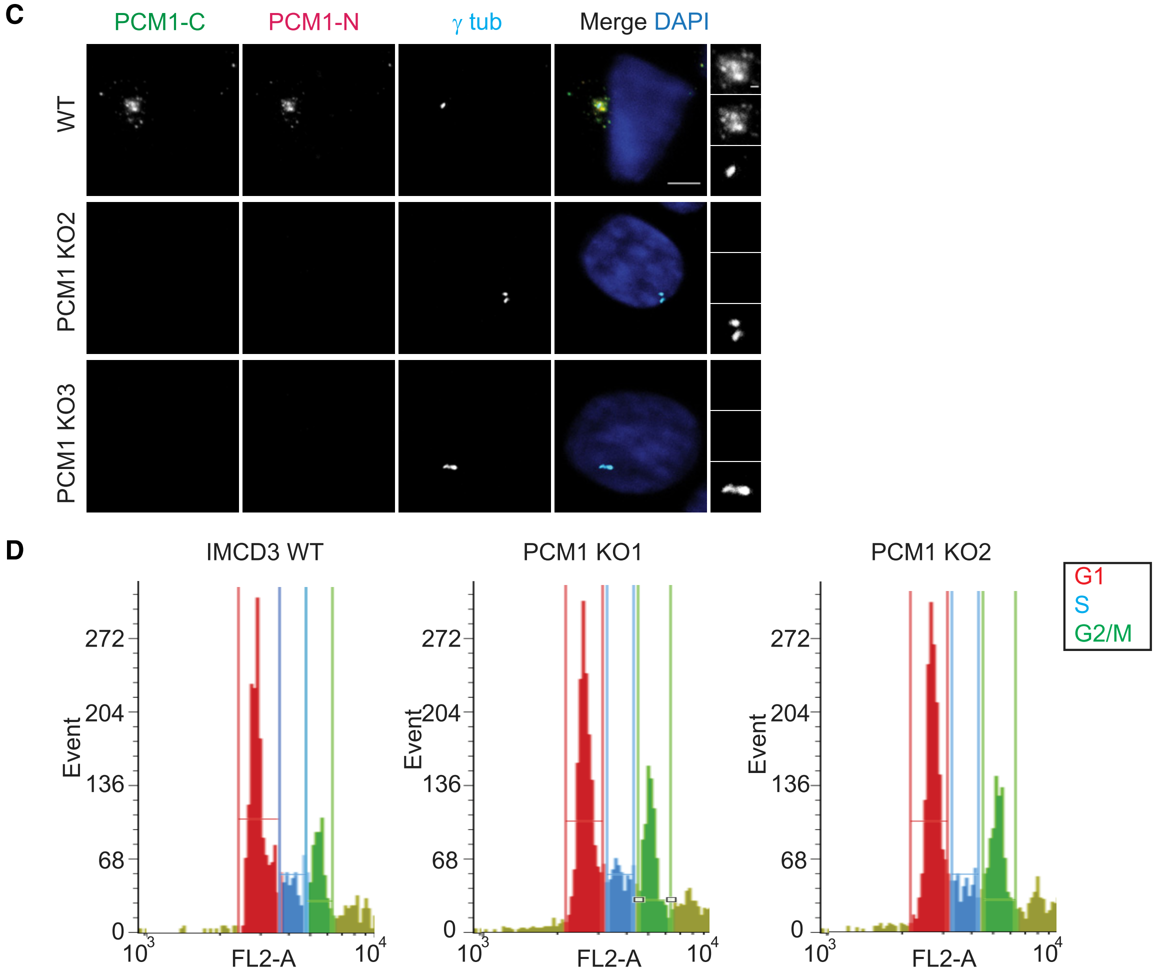

Figure EV1.

 © 2019 The Authors

Figure EV2. Satellite-less RPE1 cells are defective in cilium formation, but not in cell cycle progression, proliferation, and centriole duplication.

A RPE1 PCM1 KO clones are all compound heterozygotes with mutations that lead to early stop codons. 1,000 bp region around the gRNA-target site was PCR-amplified and cloned. Sequencing of five different clones for each line identified one 1-nt insertion on one allele and one-nt insertion and one-nucleotide change on the other for line 1, 7-nt deletion on one allele and 4-nt deletion and 2-nt insertion on the other for line 2.

B Translation products of the gRNA-targeting exon (protein-coding exon 2) in RPE1 PCM1 KO clones.

C Immunoblot analysis of whole-cell lysates from control cells and two RPE1 PCM1 KO clones with PCM1 antibody targeting N-terminal 1–254 amino acids.

D Immunofluorescence analysis of control and RPE1 PCM1 KO clones. Cells were fixed and stained for centrosomes with anti-γ-tubulin antibody and PCM1 with PCM1-N antibody and PCM1-C antibody. Scale bar, 3 μm.

E Effect of satellite loss on cilium formation. Control and RPE1 PCM1 KO cells were serum-starved for the indicated times, and percentage of ciliated cells was determined by staining for acetylated tubulin, Arl13b, and DAPI. Results shown are the mean of three independent experiments ± SD (500 cells/experiment, ***$P < 0.001$, *t*-test).

F Effects of satellite loss on cell proliferation. 10^5 cells were plated and counted at 1, 2, 3, and 5 days. Data points show mean ± SD of three independent experiments. There was no significant difference between control and RPE1 PCM1 KO cells at any time point.

G Effect of satellite loss on percentage of cells in different cell cycle phases. Flow cytometry analysis of control or RPE1 PCM1 KO cells. Data points show mean ± SD of three independent experiments.

H Effect of satellite loss on centriole duplication. Asynchronous control and RPE1 PCM1 KO cells were stained with staining for centrin2, centrin3, and gamma-tubulin and DAPI. Quantification results shown are the mean of two independent experiments ± SD (100 cells/experiment, *t*-test). Scale bar, 4 μm.

© 2019 The Authors

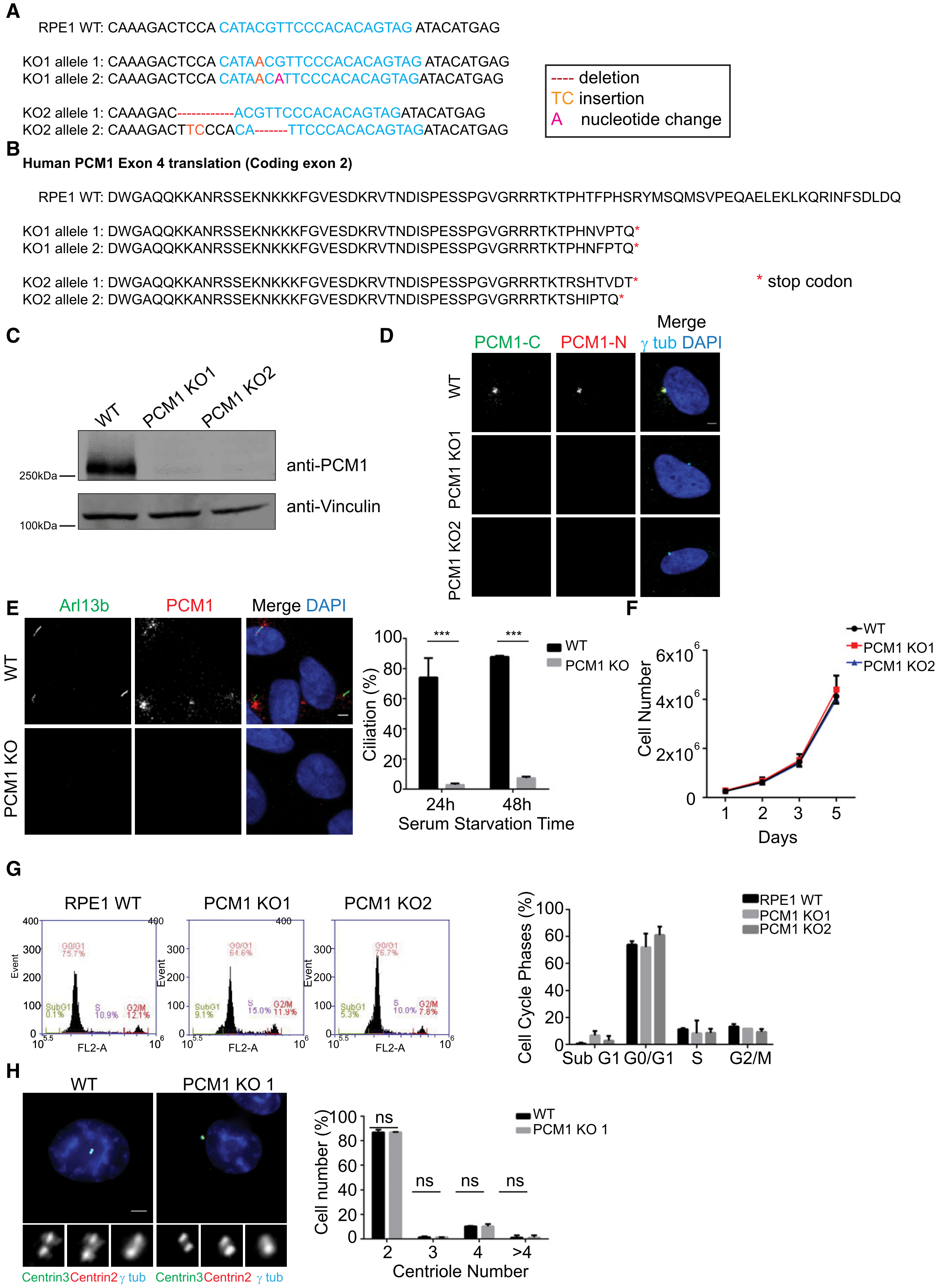

Figure EV2.

 © 2019 The Authors

Figure EV3. Centrosomal abundance of key ciliogenesis factors in asynchronous IMCD3 PCM1 KO and control cells.

A–F Effect of satellite loss on centrosomal abundance of proteins in asynchronous cells. Control, IMCD3 KO, and IMCD3 KO stably expressing LAP-PCM1 cells were fixed and stained with antibodies for the indicated proteins along with the centrosome marker anti-gamma-tubulin. Scale bar, 1 μm. Both ciliated and unciliated cells were quantified in a blinded manner. Images represent cells from the same coverslip taken with the same camera settings. The centrosomal fluorescence intensity of the indicated proteins was measured for control and IMCD3 PCM1 KO cells in a 3 μm^2 square area around the centrosome, and levels were normalized to 1. Results shown are the mean of two independent experiments ± SD (100 cells/experiment, $^{**}P < 0.01$, $^{***}P < 0.001$, $^{****}P < 0.0001$, ns: not significant, *t*-test) Error bars represent SD. Horizontal lines represent the mean value of each group and the mean of the control group was normalized to 1. The boxes include data from the 25th to 75th percentiles of each group.

G Effects of satellite loss on cellular abundance of centrosome proteins. Whole-cell lysates from asynchronous control and IMCD3 PCM1 KO cells were immunoblotted with the indicated antibodies. Vinculin was used as a loading control. Results shown are the mean of two independent experiments ± SD ($^{**}P < 0.01$, $^{***}P < 0.001$, $^{****}P < 0.0001$, *t*-test).

© 2019 The Authors

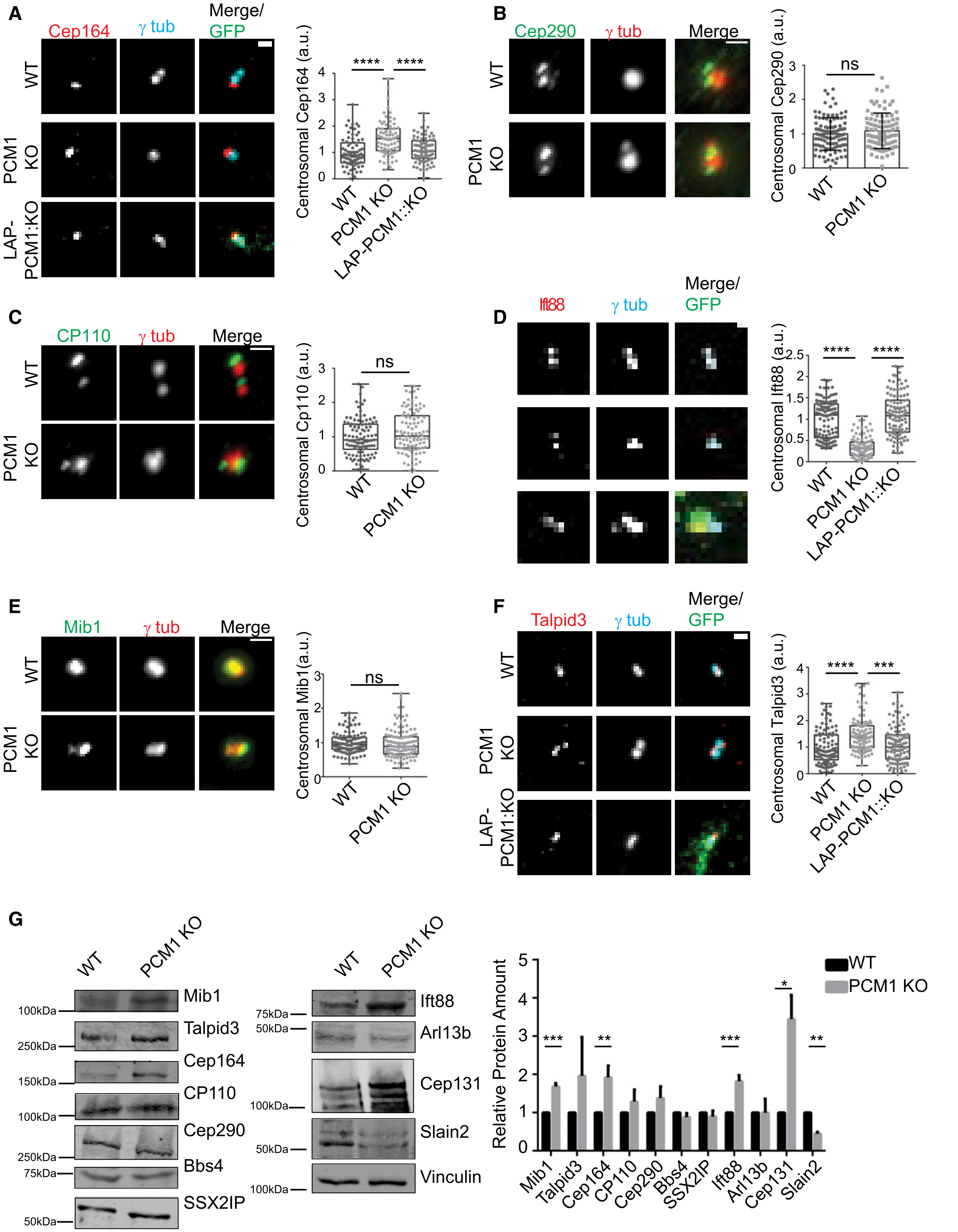

Figure EV3.

© 2019 The Authors

Figure EV4. FRAP experiments in IMCD3 control and PCM1 KO cells expressing SSTR3-LAP and HTR6-LAP.

A–D Representative still images of FRAP in the (A) whole cilium and (B) half-cilium in IMCD3 24 h after serum starvation of IMCD3 cells stably expressing SSTR3-LAP and HTR6-LAP. Representative examples of different time points from each FRAP experiments are shown. Scale bar, 1 μm. (C, D) Quantification of half-cilium FRAP experiments for control and PCM1 KO cells expressing (C) SSTR3-LAP and (D) HTR6-LAP. Kinetics of average (± SD) fluorescence recovery of proteins photobleached in the half-cilium, half-time of recovery $t^{1/2}$, and percentage of recovery were quantified (n = 5 cells).

© 2019 The Authors

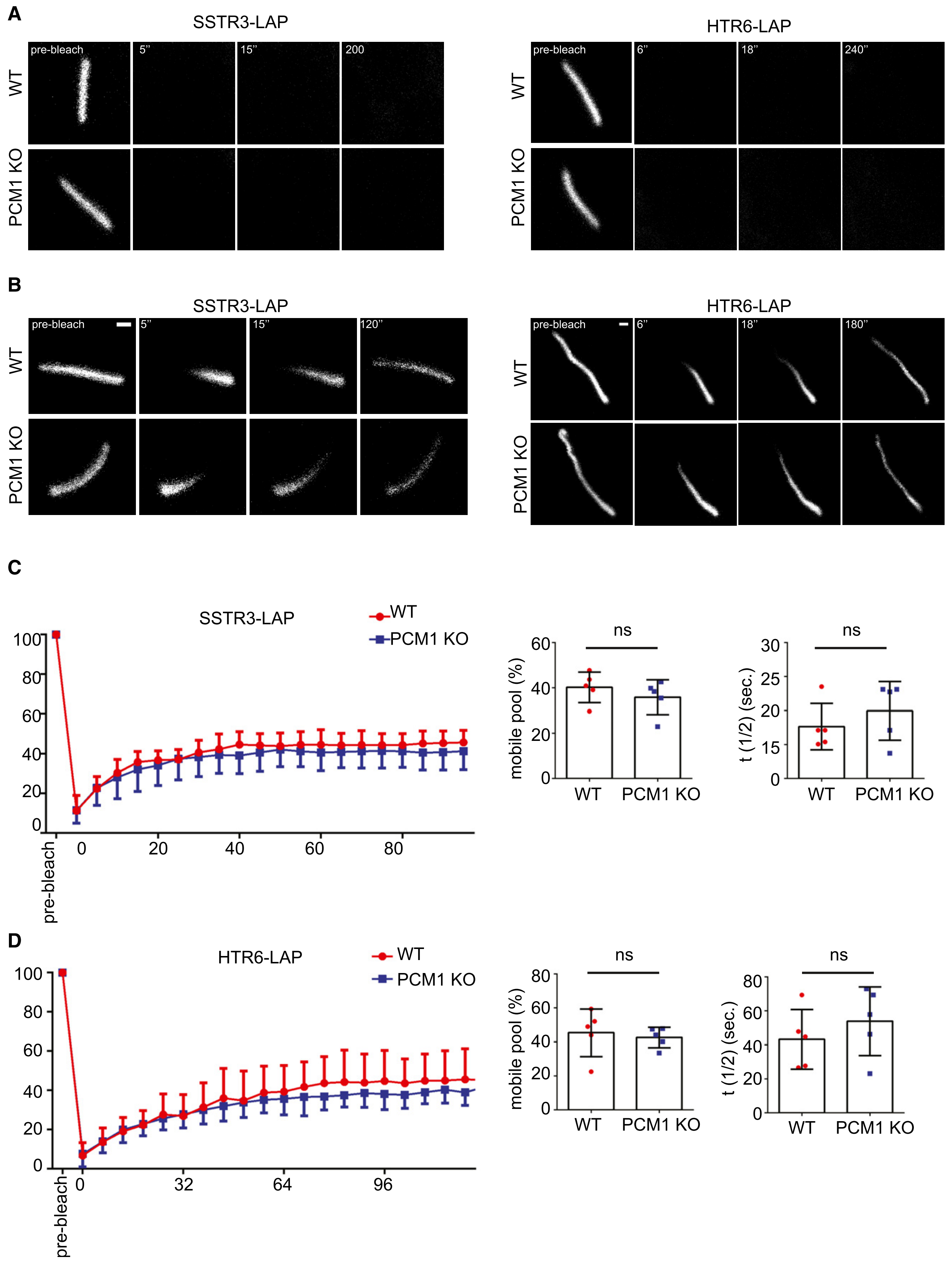

Figure EV4.

 © 2019 The Authors

Figure EV5. Interaction network and GO analysis of the upregulated and downregulated proteins in IMCD3 PCM1 KO cells.

A, B Interaction network analysis for (A) downregulated and (B) upregulated proteins identified by GO-enrichment analysis revealed pathways enriched or depleted in satellite-less cells.

C, D GO-enrichment analysis for upregulated and downregulated proteins in satellite-less cells in (C) biological function and (D) cellular compartment categories.

© 2019 The Authors

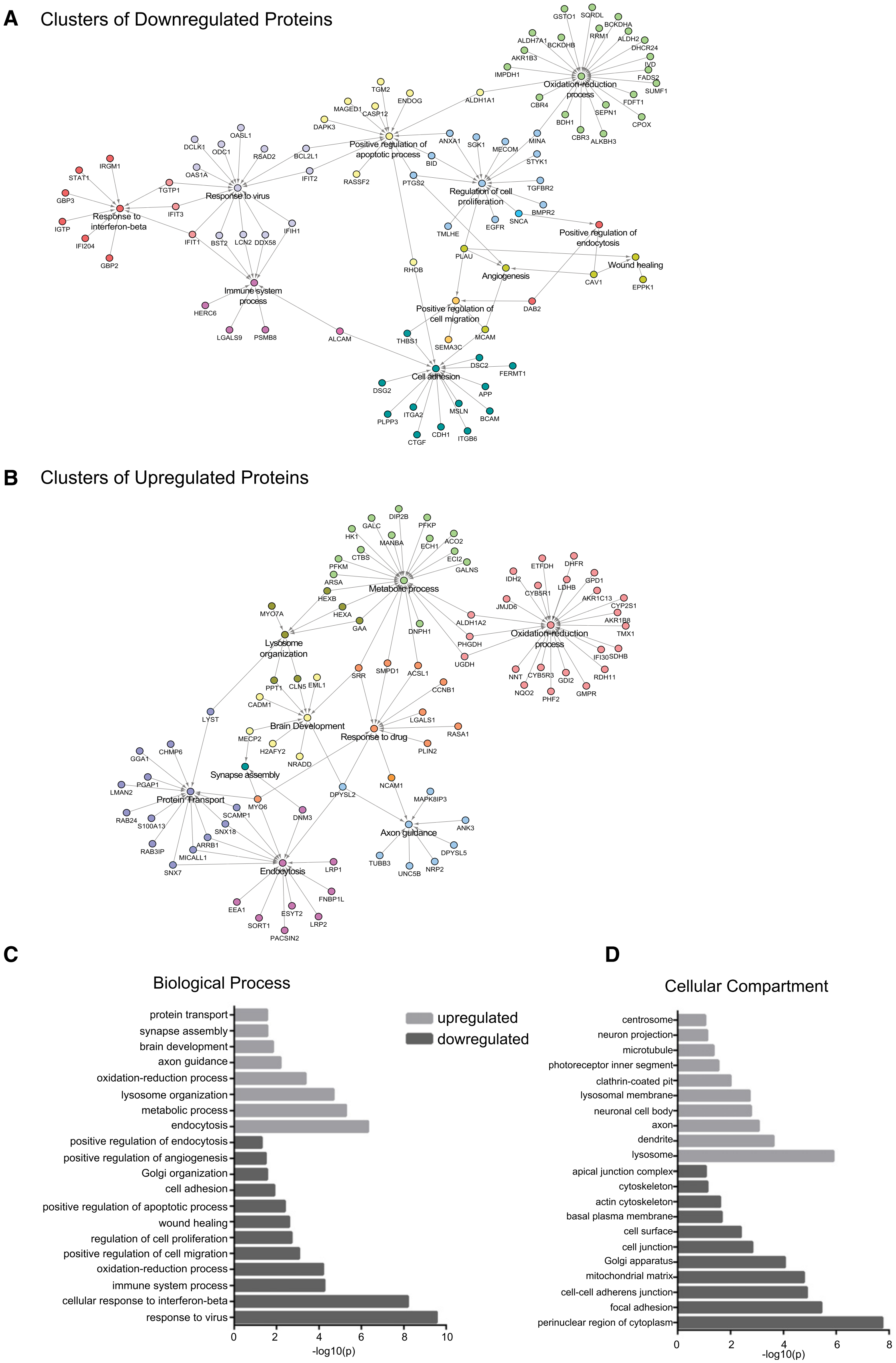

Figure EV5.

 © 2019 The Authors
